# A novel cardiac muscle-derived biomaterial reduces dyskinesia and postinfarct left ventricular remodeling in a mouse model of myocardial infarction

**DOI:** 10.14814/phy2.12351

**Published:** 2015-03-29

**Authors:** Daniel M O'Connor, Nivedita K Naresh, Bryan A Piras, Yaqin Xu, Robert S Smith, Frederick H Epstein, John A Hossack, Roy C Ogle, Brent A French

**Affiliations:** 1 Department of Biomedical Engineering, University of VirginiaCharlottesville, Virginia; 2 Department of Radiology, University of VirginiaCharlottesville, Virginia; 3 School of Medical Diagnostic and Translational Sciences, College of Health Sciences, Old Dominion UniversityNorfolk, Virginia

**Keywords:** Extracellular matrix, left ventricular remodeling, myocardial infarction, small animal imaging, speckle tracking echocardiography

## Abstract

Extracellular matrix (ECM) degradation after myocardial infarction (MI) leaves the myocardium structurally weakened and, as a result, susceptible to early infarct zone dyskinesia and left ventricular (LV) remodeling. While various cellular and biomaterial preparations have been transplanted into the infarct zone in hopes of improving post-MI LV remodeling, an allogeneic cardiac muscle-derived ECM extract has yet to be developed and tested in the setting of reperfused MI. We sought to determine the effects of injecting a novel cardiac muscle-derived ECM into the infarct zone on early dyskinesia and LV remodeling in a mouse model of MI. Cardiac muscle ECM was extracted from frozen mouse heart tissue by a protocol that enriches for basement membrane constituents. The extract was injected into the infarct zone immediately after ischemia/reperfusion injury (*n* = 6). Echocardiography was performed at baseline and at days 2, 7, 14, and 28 post-MI to assess 3D LV volumes and cardiac function, as compared to infarcted controls (*n* = 9). Early infarct zone dyskinesia was measured on day 2 post-MI using a novel metric, the dyskinesia index. End-systolic volume was significantly reduced in the ECM-treated group compared to controls by day 14. Ejection fraction and stroke volume were also significantly improved in the ECM-treated group. ECM-treated hearts showed a significant (*P* < 0.005) reduction in dyskinetic motion on day 2. Thus, using high-frequency ultrasound, it was shown that treatment with a cardiac-derived ECM preparation reduced early infarct zone dyskinesia and post-MI LV remodeling in a mouse model of reperfused MI.

## Introduction

In its acute stage, myocardial infarction (MI) results in immediate death to cardiomyocytes and contractile dysfunction. Immune cells infiltrate the area of infarction to phagocytize necrotic cells and apoptotic cell debris, and release enzymes that degrade the cardiac extracellular matrix (ECM) (Dai et al. 2005; French and Kramer 2007; Jourdan-Lesaux et al. 2010). This causes the infarct zone to become thinned, structurally weak, and susceptible to paradoxical bulging under the pressure of systole (early dyskinesia) (Dai et al. 2005; French and Kramer 2007; Gersh et al. 2009) prior to scar formation. Early dyskinesia not only robs the heart of systolic function, but is also correlated with maladaptive long-term changes in heart morphology and function, and thus may contribute importantly to left ventricular (LV) remodeling and eventual heart failure (French and Kramer 2007).

Although a number of clinical studies have shown that the transplantation of progenitor or stem cells into the LV after MI can marginally improve cardiac function, the data from most such studies fail to show clinically significant levels of differentiation (much less survival) of the transplanted cells (Gersh et al. 2009). These data suggest that the major benefits of cell transplantation therapy may not result from replacing lost cardiomyocytes, but rather from the injected cells serving to reinforce the infarcted LV walls via a scaffolding effect (Dai et al. 2005) or by producing cytokines and growth factors that stimulate regeneration (Gnecchi et al. 2005).

These current hypotheses surrounding stem cell transplantation therapy support a strategy involving acellular therapy. ECM components are an attractive material for acellular therapy because they serve a structural function and promote cellular adhesion, proliferation, and differentiation (Akhyari et al. 2008). Transplanted ECM derived from porcine urinary bladders has been found to become populated with myocytes and improve regional contractile function in several animal models (Kelly et al. 2009; Singelyn and Christman 2010). ECM composition is complex, however, and highly variable among species and cell types and the composition of the extract will almost certainly determine its properties. Recently, a hydrogel derived from porcine cardiac ECM was found to preserve cardiac function in a rat MI model (Singelyn et al. 2012).

Matrigel (BD Biosciences, San Jose, CA), a reconstituted basement membrane-rich extract, has been widely used as a cell culture substrate for over 30 years (Kleinman et al. 1986). While Matrigel has been implanted to treat postinfarct myocardium, it is derived from a mouse sarcoma and is thus comprised of noncardiac matrix components and is potentially immunogenic outside the mouse, limiting its clinical potential (Singelyn and Christman 2010). The biochemical protocol used in Matrigel production can be adapted, however, to other basement membrane-rich tissues such as skeletal muscle (Abberton et al. 2008).

This study sought to determine whether a novel cardiac muscle-derived ECM extract (“cMatrix”) could improve cardiac function after reperfused MI. In order to test the therapeutic potential of cMatrix, high-frequency 3D echocardiography was used to serially assess cardiac function over 28 days in a mouse model of reperfused MI. Cardiac magnetic resonance (CMR) was performed in a subset of mice on day 2 post-MI to determine infarct size, and on day 28 post-MI to confirm volumetric results. A new metric, here termed the “dyskinesia index” (DI), was developed to measure early infarct zone dyskinesia, based on wall-motion data acquired from ultrasound image processing.

## Materials and Methods

### Experimental design

**Figure 1:**
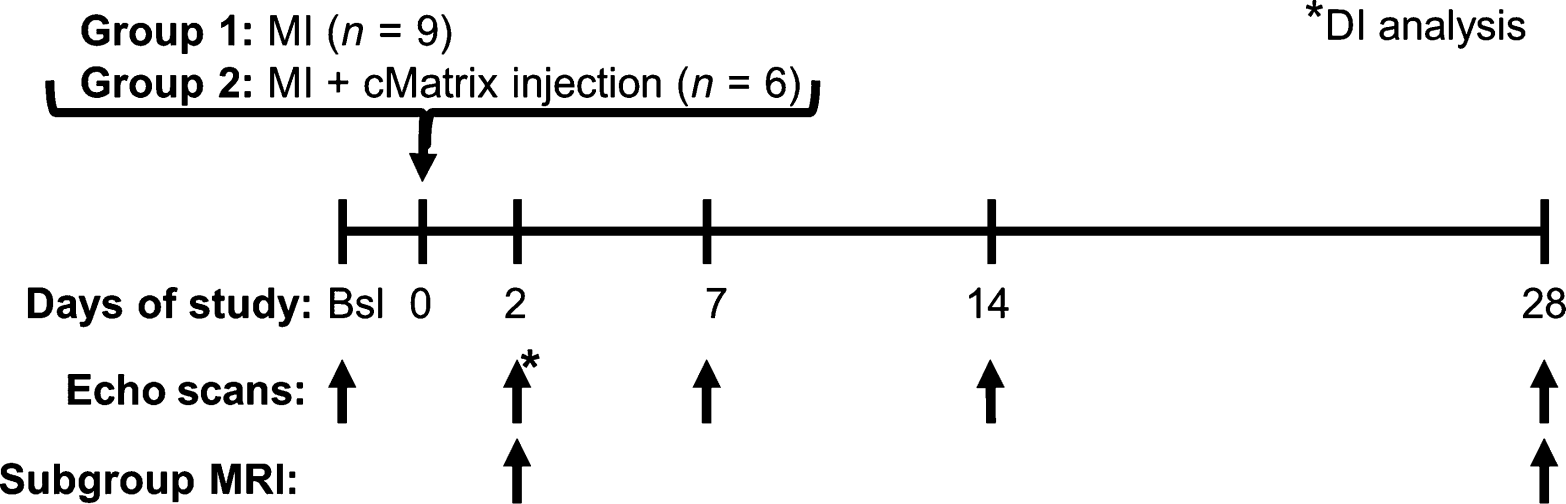
Experimental Design. Fifteen mice received a 1-h coronary occlusion followed by reperfusion on day 0 (MI). Nine of these mice were left untreated, while six received cMatrix injection at reperfusion. All mice were studied using 3D echocardiography, both at baseline and at 2, 7, 14 and 28 days post-MI. DI analysis was performed using data collected on day 2 post-MI. MRI scans were performed in a subset of mice on day 2 post-MI to determine infarct size, and on day 28 post-MI to confirm volumetric results.

The experimental design of this study is summarized in Figure1. 3D echocardiography was performed at baseline and on days 2, 7, 14, and 28 post-MI and involved the capture of a full sweep of short-axis (SA) cines from the apex to the base of the LV at 1.0 mm intervals. This imaging was achieved using a custom-built hardware/software system that controlled the ultrasound transducer and triggered the scanner to acquire and store data, allowing for the full SA stack to be captured in less than 1 min. A novel metric based on the ratio of signed to unsigned endocardial displacement, here termed the “dyskinesia index” (DI), was employed to quantify early infarct zone dyskinetic motion on post-MI D2. MRI was performed in a subset of mice on D2 post-MI to determine infarct size, and on D28 post-MI to confirm volumetric results.

### Tissue harvesting for cMatrix

This study was conducted under protocols approved by the University of Virginia Animal Care and Use Committee. Adult male C57Bl/6 mice purchased from The Jackson Laboratory (Bar Harbor, ME) were killed with an overdose of anesthetic and death was confirmed by cervical dislocation. Hearts were then immediately excised, rinsed in chilled phosphate-buffered saline (PBS), and atria were removed. The tissue was then snap-frozen in liquid nitrogen and stored at −80°C.

### cMatrix production

All procedures were performed at 4°C. Frozen mouse tissue was thawed from −70°C in buffered 3.4 M NaCl with added protease inhibitors [5 mM ETDA, 2 mM NEM (Sigma-Aldrich, St. Louis, MO)], in a volume six times the weight of the sample. The sample was minced into approximately 1 mm^3^ pieces using a scalpel and homogenized. The homogenized mixture was then spun at 10,000 × g for 30 min. The pellet was washed with a 2 M buffered urea solution at a volume equal to the original weight of the tissue. The sample was then stirred overnight. Next, the sample was centrifuged at 18,000 × g for 30 min and the supernatant was decanted and stored at 4°C. Two additional extractions were performed by resuspending and homogenizing the pellet in urea buffer of one-half the volume of the original tissue weight, and repeating the centrifugation. Aliquots from each sample were evaluated for total protein and collagen content to assess quality. The combined supernatants were then dialyzed against 0.5% chloroform in tris-buffered saline (TBS) overnight to sterilize the extract. The extract was further dialyzed twice against TBS to remove the urea and chloroform, and once against PBS to enhance physiological compatibility. The final extract was aliquoted and stored at −20°C.

### Protein concentration measurements

Total protein concentration was measured using an assay kit based on the Lowry method (Bio-Rad, Hercules, CA). Collagen content was determined using the Sircol soluble collagen assay kit (Biocolor; Accurate Chemical & Scientific Corporation, Westbury, NY).

### Mouse model of reperfused MI

Male C57Bl/6 mice were purchased from Jackson Laboratories (Bar Harbor). The mice were between 10–12 weeks old and weighed 24–29 g at the time of surgery. A standard myocardial ischemia/reperfusion protocol was employed, as described previously (Li et al. 2011). Briefly, the hearts of anesthetized mice were exposed via left thoracotomy, and occlusion was induced by passing a suture around the left anterior descending coronary artery and tightening it over a piece of polyethylene-60 tubing for 60 min. For the group of mice receiving cMatrix, 15 *μ*L were injected directly into the anterolateral sector of apical myocardium using a 31-gauge insulin syringe.

### Echocardiography

All mice were studied by echocardiography at baseline and 2, 7, 14, and 28 days after myocardial infarction (MI). Prior to scanning, fur was shaved from the chest followed by depilation with Nair cream. Ultrasound B-mode cardiac image sequences were acquired using a Vevo 2100 high-resolution ultrasound scanner (VisualSonics Inc., Toronto, ON, Canada). Anesthesia, body temperature, and EKG monitoring were performed as described previously (Li et al. 2011).

Six to seven serial parasternal left ventricular (LV) short-axis (SA) views were captured from the apex to the base of the LV in 1.0 mm intervals using a custom-built hardware/software system that controlled the motor stage holding the transducer and triggered the scanner to acquire and store image data. This system allowed for the full SA stack to be captured in less than one minute. Sequences averaged 30–40 frames per cardiac cycle. The total ultrasound imaging session duration was under 30 min per mouse.

Left ventricular short-axis cross-sectional areas were calculated by manually delineating endocardial contours at end-diastole (ED) and end-systole (ES) at 1 mm intervals from apex to base. ED and ES were determined on a per slice basis. The LV end-systolic volumes (LVESV) and LV end-diastolic volumes (LVEDV) were then computed, and ejection fraction (EF) and stroke volume (SV) were derived from these values.

The extent of wall thinning was calculated from endocardial and epicardial traces of long-axis images at end-diastole. Significant wall thinning was defined as a left ventricular wall thickness of 0.5 mm or thinner, relative to the normal end-diastolic wall thickness of 1 mm. The extent was defined as the percentage of the LV wall in which significant wall thinning occurred.

### Echocardiography data analysis

A novel metric, here termed the “dyskinesia index” (DI) was employed to quantify early infarct zone dyskinetic motion, based on contraction index, as described by Li et al. (2011). DI is a measure based on the efficiency (eff) of endocardial displacement, which is defined as the ratio of the sum of signed radial displacements to the sum of unsigned radial displacements for each time point in the cardiac cycle: 

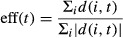
1

In this equation, “*d*” is the mean radial displacement of each segment “*i*”, at time point “*t*”. DI is defined as the average value of eff*(t)* over the course of the cardiac cycle (from *t*_0_ to *t*_N_): 

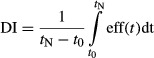
2

Thus, DI represents the average ratio of the sum of the signed radial displacements to the sum of the unsigned radial displacements, over the cardiac cycle. DI yields possible values ranging from 0 to 1, where a DI of unity represents completely polar radial displacement, and smaller values indicate increasing dyskinetic motion.

In order to measure early infarct zone dyskinetic motion, DI was calculated on D2 post-MI using a short-axis slice 2 mm superior to the apex of the LV. VevoStrain speckle tracking software was used to obtain endocardial radial displacements for 96 segments over the course of a cardiac cycle (VisualSonics Inc.) and DI was calculated as defined by Equation 2.

### MRI

MRI was performed in a subset of mice on D2 post-MI to determine infarct size, and on D28 post-MI to confirm volumetric results using previously described methods on a 7T MRI scanner (Bruker, Ettlingen, Germany) (Helm et al. 2008). Infarct sizes were determined in cMatrix-treated (*n* = 3) and infarcted control (*n* = 6) mice on day 2 post-MI using late gadolinium-enhanced (LGE) CMR images. Infarct size was expressed as a percentage of the total LV mass. Post-MI day 28 volumetrics were determined for 3 cMatrix-treated mice using a black blood gradient echo pulse sequence imaging as described previously (Helm et al. 2008). A set of six to eight contiguous SA cine images were then acquired to cover the entire LV.

### Tissue harvesting and histology

At D29 post-MI, the mice were killed with an overdose of anesthetic, and death was confirmed by cervical dislocation. Hearts were immediately excised and washed in PBS. The apical half of each heart was fixed in paraformaldehyde overnight at 4°C. The tissue was then washed three times in PBS and stored in 70% ethanol prior to embedding in paraffin and sectioning (5 *μ*m thickness). Tissue sections were then stained with picrosirius red to detect collagen.

### Statistical analysis

All data are presented as mean ± SEM. Statistical significance was presumed where *P* < 0.05. Data collected during reperfusion (days 2–28) were analyzed using two-way ANOVA for repeated measures. Post hoc analyses (Bonferroni post tests) were performed where appropriate. All other statistical analysis was performed using a two-tailed, unpaired Student's t-test. Statistical analyses were performed using Minitab version 16.1.1 (Minitab Inc, Pennsylvania State University).

## Results

### cMatrix composition

Using the Lowry total protein assay, cMatrix had a total protein concentration of 5.1 mg/mL. Concentration of collagen types I through V was 240 mg per mg of total protein. Both the total protein and collagen concentration of cMatrix are comparable to the levels reported in similar extracts, such as Myogel and Matrigel (Kleinman et al. 1986; Abberton et al. 2008). The composition of cMatrix is likely most similar to that of the skeletal muscle-derived Myogel, which contains a mixture of predominately Type I and Type IV collagens, in addition to laminins, proteoglycans, and growth factors.

### LV remodeling and cardiac function

The LV remodeling study included 9 control and 6 cMatrix-treated mice subjected to 60 min of ischemia followed by reperfusion. Mortality during surgery and over the course of the imaging period was similar in control and cMatrix-treated groups: one mouse from the control group died on day 3 post-MI after a poor recovery from surgery, and a cMatrix-treated mouse had to be killed during the last week of the study due to inadvertent injury. Volumetric data are reported relative to the baseline LV volumes for each group due to a small, but statistically significant, difference in baseline LV end-diastolic volume (LVEDV) between groups.

**Figure 2:**
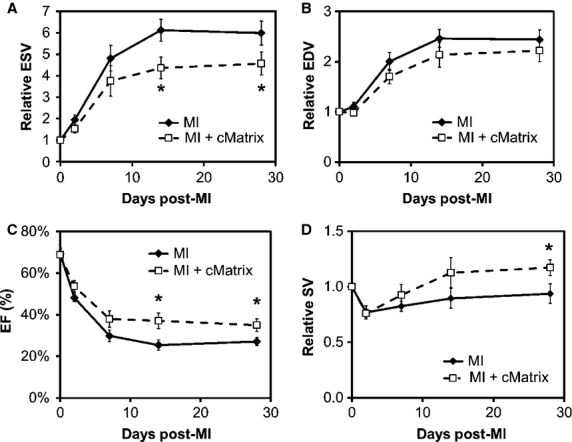
cMatrix treatment helps to preserve cardiac function and inhibits post-MI LV remodeling. (A) Relative LVESV was significantly reduced in the cMatrix-treated group (*n* = 6), compared to controls (*n* = 9) by day 14 post-MI. (B) Relative LVEDV was reduced at each time point measured over 28 days post-MI, although this trend failed to achieve statistical significance. (C) EF in the cMatrix group was significantly improved over the untreated group at days 14 and 28 post-MI. (D) By day 28, SV was significantly improved in the cMatrix-treated group. *P < 0.05 vs. untreated, infarcted control group at the same time point.

Serial 3D echocardiography performed at baseline and on days 2, 7, 14, and 28 post-MI showed improved LV remodeling and cardiac function in cMatrix-treated mice (Fig.2). Late-gadolinium-enhanced (LGE) CMR imaging performed on day 2 confirmed no significant difference in acute infarct size as a percent of LV mass between groups (controls = 31.4 ± 1.3% of LV, cMatrix-treated = 35.4 ± 6.8% of LV; *P* = NS). Relative LV end-systolic volume (LVESV) was found to be significantly reduced in cMatrix-treated mice by day 14 and remained significant (*P* < 0.05) at day 28 (Fig.2A; results expressed as fold changes relative to baseline). On day 28, the cMatrix-treated mice showed a 28% improvement in relative LVESV compared to the control group. While there was no significant difference in LVEDV between groups (Fig.2B), LV ejection fraction (EF) was also found to be significantly improved in cMatrix versus control mice by day 14 and remained improved through day 28 (Fig.2C; *P* < 0.05 at both time points). On day 28, EF was 35.0 ± 3.0% in cMatrix-treated mice versus 27.1 ± 2.0% in controls. By day 28, relative stroke volume (SV) was 22% greater in cMatrix vs. control mice (Fig.2D; *P* < 0.05). A CMR follow-up study was performed to confirm these results in a subset of animals on day 28 (*n* = 3). No statistically significant differences in LVESV or LVEDV were found between the two modalities.

### Early infarct zone dyskinetic wall motion

**Figure 3:**
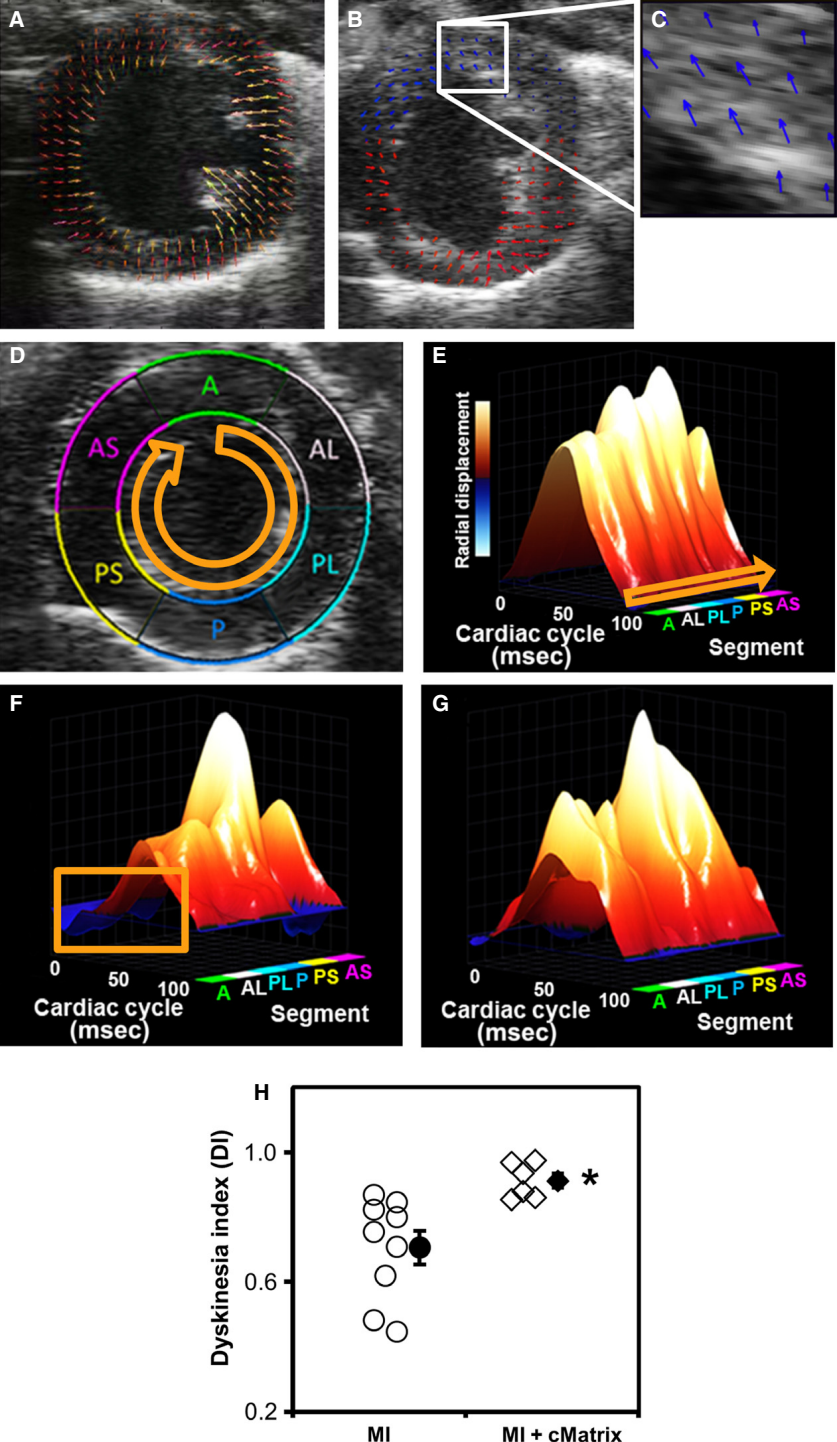
cMatrix treatment reduces early infarct zone dyskinetic wall motion. (A) A short-axis image of an uninfarcted heart in systole, with arrows representing displacement vectors. (B) A post-MI day 2 animal with severe paradoxical systolic bulging in the anterior and anterolateral segments of myocardium (white box), highlighted in “C”. (D) Standard six-sector LV short-axis (SA) segmentations are overlaid on a B-mode echocardiographic image taken 2 mm superior to the apex. “A,” anterior; “AL,” anterolateral; “AS,” anterior septum; “P,” posterior; “PL,” posterolateral; “PS,” posterior septum. “E–G” are three-dimensional representations of SA endocardial radial displacements over the course of a complete heart cycle in (E) normal, (F) day 2 post-MI untreated, and (G) day 2 post-MI cMatrix-treated mouse hearts. Dark blue regions indicate dyskinetic displacements as highlighted by the orange box. (H) cMatrix-treated hearts (*n* = 6) showed a significant improvement in dyskinesia index (DI) on day 2 post-MI compared to untreated controls (*n* = 9). A DI of unity represents completely polar radial displacement, whereas smaller values indicate increasing dyskinetic motion (*P < 0.005 vs. infarcted control).

To determine the effect of cMatrix on early infarct dyskinesia, displacement data were obtained from speckle tracking analysis of echocardiographic image series collected on day 2 post-MI from short-axis (SA) image planes positioned 2 mm superior to the apex of the heart. Figure3A shows a short-axis image of an uninfarcted heart in systole, with arrows representing displacement vectors. A post-MI day 2 animal with severe paradoxical systolic bulging in the anterior and anterolateral segments of myocardium is shown in Figure3B, with the area of dyskinetic motion highlighted in the white box, and in Figure3C. Figure3D shows a map defining the locations of the endocardial radial displacements that were determined over the course of a complete heart cycle. Figure3E–G show graphs with the SA linearized along the *y*-axis, allowing displacement data (*z*-axis) over the course of the cardiac cycle (*x*-axis) to be viewed for each SA segment in an uninfarcted mouse (Fig.3E), an untreated control mouse on day 2 post-MI (Fig.3F), and a day 2 post-MI cMatrix mouse (Fig.3G). Note that although both MI groups show deficits in contractile function in the anterolateral wall, the cMatrix group is protected from regional bulging (orange box). These findings were quantified using the novel DI metric, which was found to be significantly improved in cMatrix-treated (91.2 ± 2.2%) versus untreated hearts (71 ± 5.2%; Fig.3H, *P* < 0.005).

### Histological confirmation of cMatrix injection and reinforcement of the LV

**Figure 4:**
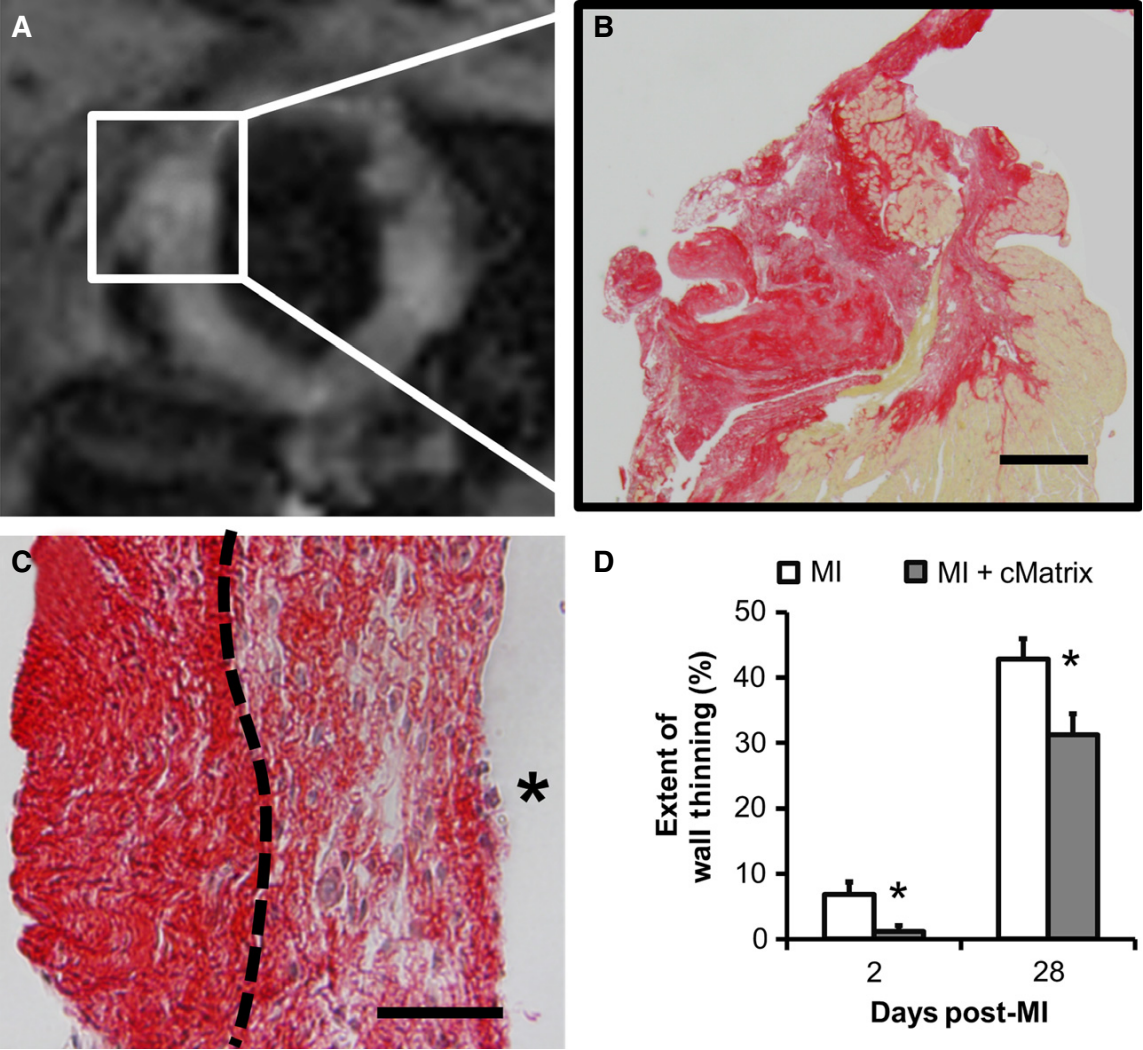
Confirmation of cMatrix injection and reinforcement of the LV. (A) An in vivo MRI short-axis (SA) image of a day 28 post-MI heart treated with cMatrix. (B) A histological slice of the boxed region (anterior septum) taken from the same animal shown in “A”. The tissue slice was stained with picrosirius red, which colors collagen red and other tissue yellow. Scale Bar = 250 *μ*m. (C) Picrosirius red stained tissue from the apical anterior wall of a cMatrix-treated mouse. The dashed line delineates the border between native tissue (right) and the relatively denser-staining, and less cellular injected matrix. The star denotes the ventricular chamber. Scale Bar = 100 *μ*m. (D) The extent of wall thinning, measured by ultrasound as the percentage of long-axis tissue with a thickness of less than 0.5 mm, was significantly decreased in the cMatrix-treated group as early as post-MI day 2. This decrease was preserved through day 28 (*P < 0.05).

The presence of cMatrix in the LV was confirmed by observing a thickening of the anterior septum surrounding the site of injection using CMR imaging on day 28 (Fig.4A) and by histological analysis (Fig.4B). Picrosirius red staining revealed a significant collagen deposit reinforcing the anteroseptal wall. Figure4C demonstrates the border (dashed line) between native collagen tissue deposition (right) and the relatively denser-staining, and less cellular injected matrix. The extent of wall thinning, measured by ultrasound as the percentage of long-axis tissue with a thickness of less than 0.5 mm on ultrasound, was significantly decreased in the cMatrix-treated group as early as post-MI day 2 (Fig.4D). This decrease was preserved on day 28 (*P* < 0.05).

## Discussion

This study describes the preparation and evaluation of cMatrix, a novel cardiac muscle-derived biomaterial. After reperfused MI, cMatrix-treated mice were shown to have reduced LV remodeling compared to infarcted control mice. Furthermore, speckle tracking displacement data were used to show that cMatrix reduced early infarct zone dyskinesia, as quantified using the novel dyskinesia index (DI).

The cMatrix extract provides a number of advantages over previously tested scaffolds. Unlike solid scaffolds or patches, liquid scaffolds are not limited to superficial placement on the epicardial surface of the heart and have the ability to fully adapt to their environment (Singelyn and Christman 2010).

Furthermore, because they can be implanted via percutaneous trans-coronary-venous or transendocardial injection, liquid scaffolds have greater clinical potential than patches, which can only be implanted surgically (Singelyn and Christman 2010). As cMatrix is an acellular therapy, it avoids risks of aberrant cellular differentiation and inadvertent cell seeding in off-target organs, as well as the potential ethical concerns associated with embryonic stem cells.

Because cMatrix is a naturally derived material, all its components are biocompatible and can be safely degraded or renewed in vivo. Additionally, synthetic compounds lack the rich combination of proteins, polysaccharides, and other ECM components that exist in a direct cardiac tissue extract such as cMatrix. Finally, cMatrix is directly derived from cardiac tissue, and therefore provides the closest possible approximation of the native cardiac environment critical to both safety and efficacy. A human-derived version of cMatrix could be produced with minimal manipulation and might well meet the FDA criteria for a Human Cell and Tissue Product (HCT/P), thus finding its way to the bedside more rapidly than many medical devices or drugs.

A potential limitation of this study is the use of untreated, infarcted mice as the negative controls, as opposed to infarcted mice injected with an equal volume of vehicle. However, we elected to use untreated, infarcted animals to better approximate the clinical situation in which patients either would receive treatment or would be left untreated. Furthermore, trauma from the needle and injected fluid might worsen cardiac function in such a vehicle control group, thus potentially overestimating the efficacy of cMatrix treatment relative to uninjected controls. A second potential limitation of this study is that it did not directly compare the injection of cMatrix to Matrigel or an alternative mixture of ECM proteins. While such alternative tissue extracts or ECM mixtures might well be successful in attenuating LV remodeling (Dai et al. 2005; Singelyn et al. 2012), one can predict that the power of such a comparative study would have to be much greater, involving many more animals, in order to capture a statistical difference between two or more materials.

The cardiac matrix preparation is a complex solution containing ECM and cardiac muscle—in essence a liquefied tissue product. Some elements of the extract promote cardiac regeneration and others appear to serve as a biocompatible delivery vehicle. Although it is of scientific importance to identify and understand which key components of the extract support regenerative activity, there is also value inherent in the simplicity of this biomaterial. The Food and Drug Administration employs the concept of minimal manipulation in classifying therapeutic agents produced from cells and tissues. The cardiac matrix described here is minimally manipulated and, arguably, could be available for clinical use as a human or cell product far sooner than a purified, manufactured product representing only the “active” ingredients in an approved carrier/delivery system. In the latter case, as a medical device or PMA, the FDA would require clinical trials, adding significantly to the time to bring the potential therapy to the patient.

Early dyskinesia after MI has previously been shown to contribute to LV remodeling (French and Kramer 2007). Indeed, our laboratory has previously demonstrated that the extent of this early paradoxical wall motion is an extremely strong predictor of subsequent LV remodeling (Li et al. 2011). This relationship may be the result of repeated cycles of systolic bulging and abnormal loading conditions causing infarct expansion and leading to positive feedback between LV remodeling and cardiac dysfunction in the subacute period prior to the formation of mature scar (Mann and Bristow 2005; French and Kramer 2007). In this study, we demonstrate that injection of cMatrix reduced early infarct zone dyskinesia independent of infarct size and attenuated LV remodeling, compared to infarcted controls. This effect could be seen as early as post-MI day 2, prior to the development of significant differences in systolic function and chamber sizes. This time point is prior to the onset of significant infarct expansion, scar formation, and other regenerative processes, such as neovascularization. It is therefore likely that cMatrix reduced LV remodeling at least partially through this direct mechanical mechanism.

The importance of early reinforcement could be further tested by assessing LV remodeling after delivering cMatrix at later time points post-MI. If early reinforcement is critical to the inhibition of LV remodeling, it is conceivable that targeting genes that control early ECM degradation (e.g., MMPs or TIMPs) may also have therapeutic effects on LV remodeling. This strategy would become particularly attractive with the advent of new gene delivery systems that rapidly and selectively target the infarct border zone after reperfused MI (Konkalmatt et al. 2012).

The nonstructural effects of cMatrix on LV remodeling remain to be examined, but are likely to be highly complex, in part because ECM composition over the course of LV remodeling is a dynamic and delicate balance of degradation and synthesis (French and Kramer 2007; Akhyari et al. 2008). In addition, various components of the ECM may have different effects on LV remodeling depending on their relative abundance and surrounding environment. For example, fibronectin fragments stimulate ECM degradation, but have also been shown to prevent apoptosis in injured cardiomyocytes (Arslan et al. 2011). Furthermore, extracts rich in basement membrane, such as cMatrix, have been shown to promote angiogenesis (Kleinman and Martin 2005). While the primary focus of this study was on the early mechanical effects of the cMatrix injection, a more detailed analysis of the effect of cMatrix on endogenous ECM synthesis and degradation post-MI could potentially shed light on additional mechanisms behind the treatment's effects on remodeling.

Much of the complex role of ECM in LV remodeling remains to be defined. Analysis and selective modification of cMatrix content may yield insight into the factors and mechanisms contributing to cardiac regeneration, paving the way for other novel strategies for curtailing LV remodeling post-MI.
